# Incidence and Predictors of Urethral Stricture Following Transurethral Resection of the Prostate and Open Simple Prostatectomy: A 21-Year Retrospective Cohort Study

**DOI:** 10.3390/jcm14113777

**Published:** 2025-05-28

**Authors:** Dor Golomb, Meitar Atias, Hanan Goldberg, Asaf Shvero, Yuval Kozlov, Yishai H. Rappaport, Orit Raz

**Affiliations:** 1 Department of Urology, Samson Assuta Ashdod University Hospital, 7 Harefua St., Ashdod 7747629, Israel; 2 Clalit Health Services, Central District, Rishon LeTsiyon 7528809, Israel; 3 Department of Military Medicine, Faculty of Medicine, The Hebrew University, Jerusalem 9190500, Israel; 4 Department of Urology, State University of New York Upstate Medical University, Syracuse, NY 13066, USA; 5 Department of Urology, Sheba Meical Center, Ramat Gan 5262000, Israel

**Keywords:** LUTS, BPH, TURP, OSP, urethral stricture

## Abstract

**Background/Objectives:** To assess the incidence and factors associated with urethral stricture following monopolar transurethral resection of the prostate (mTURP) and open simple prostatectomy (OSP) over a 21-year period. **Methods:** We conducted a retrospective cohort study of adult male patients insured by Clalit Health Services in Israel, who underwent either mTURP or OSP at multiple centers. Key baseline characteristics, including age, body mass index (BMI), socioeconomic status, Charlson comorbidity index score, and the incidence of urethral stricture, were collected. Postoperative urethral strictures were identified using the ICD-10 code N35.9 (urethral stricture, unspecified). **Results:** Between January 2000 and December 2021, 54,872 patients underwent simple prostatectomy across 29 hospitals, with 43,525 (79%) undergoing mTURP and 11,347 (21%) undergoing OSP. The median age of patients undergoing mTURP was 73.6 years, while those undergoing OSP had a median age of 72.1 years (*p* < 0.0001). The incidence of urethral strictures was 1.15% (500) following mTURP and 0.538% (61) following OSP, with an incidence rate ratio (IRR) of 2.139 (*p* < 0.0001). On multivariable analysis, factors associated with the development of urethral stricture included the type of procedure (HR = 2.349, 95% CI: 2.081–2.653, *p* < 0.0001), older age at surgery (HR = 1.012, 95% CI: 1.007–1.018, *p* < 0.0001), higher Charlson Index score (HR = 1.128, 95% CI: 1.109–1.148, *p* < 0.0001), and lower BMI (HR = 0.990, 95% CI: 0.982–0.999, *p* = 0.027). **Conclusions:** Our study highlights a higher incidence of urethral stricture following mTURP compared to OSP. Additionally, older age and a higher Charlson comorbidity index were associated with increased risk of stricture development postsurgery.

## 1. Introduction

Lower urinary tract symptoms (LUTS) in men are frequently associated with benign prostatic hyperplasia (BPH), particularly as these symptoms tend to increase in prevalence with advancing age. BPH is a common, non-malignant condition marked by the progressive enlargement of the prostate gland. The pathogenesis of BPH is multifactorial, involving a complex interplay of hormonal imbalances, genetic predispositions, aging-related tissue remodeling, and environmental influences. While BPH is not cancerous, its clinical impact can be substantial. The growing prostate tissue can compress the urethra, leading to bothersome symptoms such as weak urinary stream, increased urinary frequency, nocturia, and incomplete bladder emptying. These symptoms can diminish a patient’s quality of life and, in more severe cases, may require pharmacological management or surgical intervention to alleviate urinary obstruction and prevent further complications.

The initial management of BPH typically involves pharmacologic treatment, with surgical options considered when symptoms persist or complications develop. Medical therapy commonly includes alpha-adrenergic antagonists, which help relax prostatic and bladder neck smooth muscle to improve urine flow, and 5-alpha-reductase inhibitors (5ARIs), which reduce prostate volume over time by inhibiting the conversion of testosterone to dihydrotestosterone. Despite the efficacy of these medications, some patients may experience inadequate symptom relief or develop adverse effects, prompting consideration of surgical intervention. Simple prostatectomy is generally reserved for patients with severe BPH-related complications or when conservative measures fail. Indications for surgery include recurrent urinary retention unresponsive to catheterization, evidence of renal insufficiency secondary to bladder outlet obstruction, persistent or recurrent macroscopic hematuria attributable to BPH, recurrent urinary tract infections, formation of bladder stones, and patient preference after informed discussion of treatment options. In such cases, surgical management can provide significant and lasting relief of obstructive lower urinary tract symptoms and help prevent further morbidity [1].

TURP remains one of the most widely performed surgical treatments for BPH, offering effective symptom relief and improved urinary flow. TURP can be performed using either monopolar or bipolar electrosurgical systems, with key differences in technique and associated risks. mTURP, the traditional approach and long regarded as the gold standard, utilizes a monopolar current requiring an electrolyte-free irrigation solution such as glycine or mannitol. While effective, the use of non-conductive irrigation fluids in mTURP carries the risk of fluid absorption and the potential development of transurethral resection (TUR) syndrome—a rare but serious complication characterized by fluid overload, hyponatremia, and neurological symptoms. In contrast, bipolar TURP (bTURP) employs a bipolar electrical current that allows the use of isotonic saline as the irrigant. This not only minimizes the risk of TUR syndrome but also enhances procedural safety. Moreover, bTURP has demonstrated additional advantages, including reduced intraoperative blood loss, shorter postoperative catheterization times, and potentially shorter hospital stays. Despite these differences, both techniques have been shown to be highly effective in relieving bladder outlet obstruction and improving lower urinary tract symptoms. The surgical approach in either technique involves insertion of a resectoscope through the urethra, allowing the surgeon to visually identify and resect hypertrophic prostatic tissue responsible for the obstruction, thereby restoring urinary flow [2]. The incidence of urethral stricture following mTURP is reported to be between 2.2% and 9.8% within six months of the procedure [3,4,5].

Historically, OSP was the primary treatment for symptomatic BPH in men with significantly enlarged prostates and has proven effective in providing long-term relief from obstructive urinary symptoms, with a low rate of retreatment [6]. OSP involves making an incision in the lower abdomen to access and remove the obstructing prostate tissue. During OSP, the inner portion of the prostate, the adenoma, obstructing the urethra is removed [1]. However, due to its higher morbidity and extended recovery period compared to minimally invasive transurethral procedures, OSP has largely been replaced in developed countries like Europe and the USA by endoscopic techniques, or in some cases, robotic-assisted surgeries [7].

Urethral strictures represent a recognized and potentially debilitating complication that can arise following various urological procedures, including TURP and OSP. These strictures typically develop as a consequence of postoperative inflammation, scarring, and fibrosis within the urethral lumen, leading to a progressive narrowing that obstructs urinary flow. The resulting anatomical constriction can give rise to a wide range of LUTS, such as a weakened or intermittent urinary stream, hesitancy, straining during voiding, a persistent sensation of incomplete bladder emptying, and increased urinary frequency or urgency. Beyond their physical manifestations, urethral strictures significantly impact patients’ overall quality of life. The chronic nature of the symptoms can lead to sleep disturbances due to nocturia, heightened anxiety and frustration, and a reduction in social participation or work productivity. Recurrent urinary tract infections and the need for repeated interventions may further compound the psychological burden. In many cases, patients report a persistent sense of discomfort and disruption to daily activities, underlining the importance of timely diagnosis and individualized management to restore both urinary function and well-being.

Urethral strictures are a significant and often underrecognized complication of urological procedures, including transurethral resection of the prostate TURP and OSP. This fibrotic narrowing of the urethra can arise due to direct mechanical trauma, ischemia, inflammation, or infection, leading to substantial morbidity. Clinically, strictures can result in progressive LUTS, such as weak urinary stream, straining, incomplete emptying, and urinary retention. Beyond their impact on urinary function, strictures are associated with recurrent urinary tract infections, the need for repeated surgical interventions, and deterioration in quality of life. The burden of urethral strictures is further compounded by the potential for delayed diagnosis and the challenges associated with definitive management. Given the high prevalence of surgical treatment for BPH and the substantial long-term implications of stricture disease, understanding the incidence and predictors of this complication is essential for optimizing patient outcomes and tailoring surgical decision-making.

The data in our study, spanning 21 years, reflect a period when OSP was still more commonly performed. The most frequent late complication associated with OSP was bladder neck stenosis, occurring at a rate of 3.3% to 5.3%, while the incidence of stricture disease remained low at 0.6–2.1% [8,9]. Our study also examined the incidence of urethral strictures following both mTURP and OSP during this time.

## 2. Materials and Methods

With approval from the institutional review board (protocol number COM-0077-22), we conducted a retrospective, population-based, multicenter cohort study to evaluate postoperative outcomes in adult male patients undergoing surgical treatment for LUTS. The study included men aged 18 years and older, all of whom were insured by Clalit Health Services, Israel’s largest healthcare provider. Eligible participants underwent either mTURP or OSP between 1 January 2000, and 31 December 2021. Comprehensive baseline data were collected for each patient, including age, BMI, socioeconomic status, and comorbidity burden as assessed by the Charlson comorbidity index. A key outcome of interest was the development of urethral stricture following the surgical procedure. bTURP was excluded from the study, as it was introduced later in the study timeframe and constituted only a small fraction of cases, with inconsistent documentation across centers. Postoperative urethral strictures were identified based on documented diagnoses using the International Classification of Diseases, 10th Revision (ICD-10) code N35.9, representing “urethral stricture, unspecified.” Only cases in which this diagnosis was recorded after the index surgery were included for analysis to ensure accurate attribution of the complication to the surgical intervention.

### Statistical Analysis

Descriptive statistics were reported as means with standard deviations for continuous variables and proportions for categorical variables. Group comparisons were made using *t*-tests and chi-squared tests. Cox proportional hazards models were used to assess the impact of covariates on stricture development, adjusting for relevant factors. All analyses were two-tailed with a significance level of *p* < 0.05, performed using RStudio, version 4.1.2.

## 3. Results

**Figure 1:**
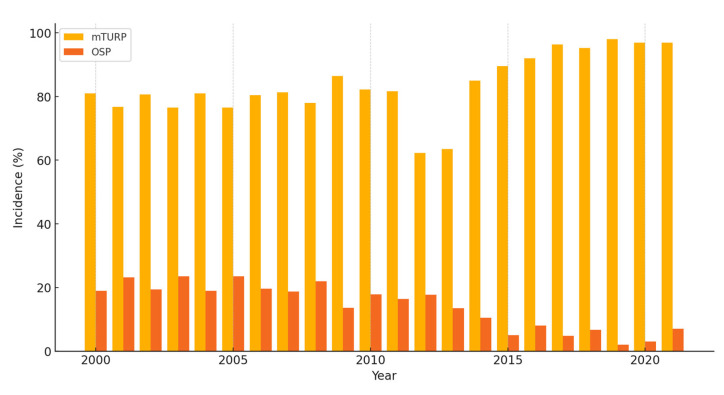
Incidence of urethral strictures by surgical modality between 2000 and 2021. mTURP—monopolar transurethral resection of prostate; OSP—open simple prostatectomy.

From 2000 to 2021, 54,872 patients underwent surgical intervention for BPH across 29 hospitals. Of these, 43,525 (79%) underwent mTURP, while 11,347 (21%) underwent OSP. Demographic and clinical data are described in Table 1. The mean ages for mTURP and OSP patients were 73.5 ± 9.36 and 72.9 ± 8.40 years, respectively (*p* < 0.0001). There was a significant slight difference in the mean BMI between the two groups, with values of 27.6 ± 4.77 for mTURP and 28.0 ± 4.91 for SOP (*p* < 0.0001). The mean Charlson comorbidity index was 5.51 ± 2.81 for mTURP patients and 4.65 ± 2.33 for OSP patients (*p* < 0.0001). The incidences of urethral strictures were 1.15 and 0.538, following mTURP and OSP, respectively, with an IRR of 2.139 (95% CI: 1.957–2.337, *p* < 0.0001). After adjusting for known confounding factors, survival analysis identified the following predictors of stricture development: the type of surgical procedure (HR = 2.349, 95% CI: 2.081–2.653, *p* < 0.0001), older age at surgery (HR = 1.012, 95% CI: 1.007–1.018, *p* < 0.0001), a higher Charlson index score (HR = 1.128, 95% CI: 1.109–1.148, *p* < 0.0001), and BMI (HR = 0.990, 95% CI: 0.982–0.999, *p* = 0.027). A summary of the crude and adjusted models is presented in Table 2. The incidence of urethral stricture was consistently higher for mTURP compared to OSP throughout the period. The difference in stricture rates between the two procedures became more pronounced in recent years, with mTURP having a significantly higher rate of strictures, particularly after 2015 (Figure 1).

## 4. Discussion

LUTS secondary to BPH stem from the gradual enlargement of the prostate gland, and typically affect aging men. This condition’s etiology involves hormonal changes, particularly an increase in dihydrotestosterone levels, leading to the proliferation of prostate glandular tissue. As a result, the enlarged prostate gland may obstruct the urethra, causing obstructive symptoms such as urinary hesitancy, weak stream, urgency, frequency, nocturia, and incomplete bladder emptying [10]. LUTS secondary to BPH significantly impact the quality of life of affected individuals [11]. Addressing these symptoms is crucial to alleviate discomfort, improve urinary function, and prevent complications such as acute urinary retention, kidney damage and urinary tract infections. Effective management strategies include medical therapy, minimally invasive procedures, and surgical interventions, which aim to restore urinary function and enhance patients’ overall well-being.

mTURP and OSP have long been cornerstone procedures in the surgical management of LUTS due to BPH. mTURP, introduced in the early 20th century, quickly became the gold standard for treating moderate to severe LUTS attributed to BPH [2]. This minimally invasive procedure involves resecting obstructive prostate tissue via the urethra. Despite advancements in medical therapy and the development of alternative treatments, mTURP remains widely used due to its proven efficacy and safety profile. On the other hand, OSP, a more invasive procedure, has been traditionally reserved for patients with significantly enlarged prostates or when mTURP was deemed insufficient or for patients with an increased risk of development of TUR syndrome, due to excessive use of glycine as an irrigation fluid and prolonged procedure time [7].

Urethral strictures, often resulting from surgical interventions, trauma, or infections, involve the narrowing of the urethra due to scar tissue formation. This condition can lead to a range of LUTS, including a weak urinary stream, straining during urination, incomplete bladder emptying, and increased frequency or urgency of urination. These symptoms can significantly impair a patient’s quality of life, causing physical discomfort, emotional distress, and social limitations. In some cases, the impact extends to family members, affecting sleep patterns and intimate relationships.

The proposed causes for the development of urethral strictures following a simple prostatectomy include infection, mechanical trauma, extended duration of catheter use, the application of local anesthesia, and electrical injury from stray currents [12,13,14]. During mTURP, the insertion and manipulation of the resectoscope, along with the resection of prostate tissue, can cause direct mechanical injury to the urethral lining. This trauma can initiate an inflammatory response, forming as the urethra heals, narrowing the urethral lumen and resulting in stricture [12,13,15]. Similarly, OSP involves significant manipulation and exposure of the urethra through an abdominal incision. We hypothesize that the procedure may lead to local ischemia, inflammation, and fibrosis through the healing process. Factors such as postoperative infections, prolonged catheterization, and patient-specific characteristics like age, comorbidities, and tissue-healing response can further contribute to the risk of stricture formation. The reported incidence of urethral stricture following mTURP ranged from 2.2 to 9.8% [3,4,5], while the incidence in OSP was 0.6–2.1% [8,9].

Our study demonstrates a significantly different incidence of urethral strictures between the mTURP and OSP groups (*p* < 0.0001), with an IRR of 2.139 (*p* < 0.0001). Multivariable analysis identified the type of surgical procedure, advanced age at the time of surgery, and a higher Charlson comorbidity index score as significantly associated with stricture development. We hypothesize that factors such as the urethral lining’s reduced ability to respond appropriately to trauma, heal effectively, and manage inflammation may contribute to stricture formation. The Charlson comorbidity index score, a widely utilized tool for predicting mortality risk by categorizing comorbid conditions, plays a crucial role in this context. Higher scores on this index reflect a greater burden of comorbidities, many of which can compromise blood flow and tissue oxygenation, induce a heightened inflammatory response, impair immune function, and increase the risk of perioperative infections. Age, which is included in the Charlson score, also emerged as being associated with stricture formation. We found a very significant difference in the incidence of urethral strictures between the mTURP group and the OSP group, as seen by the very substantial IRR. We believe this could be explained by the earlier hypothesis regarding the insertion and manipulation of the resectoscope, along with the resection of prostate tissue, causing direct mechanical injury to the urethral lining, leading to the formation of a stricture.

Interestingly, our analysis revealed a temporal trend in which the difference in urethral stricture incidence between mTURP and OSP widened over the 21-year study period, particularly after 2015. Several factors may underlie this observation. As mTURP continued to dominate the surgical landscape for BPH treatment during this time, it was increasingly performed in a broader and potentially more comorbid patient population, which may have contributed to higher complication rates, including strictures. In contrast, the number of OSP procedures declined, potentially reflecting stricter selection criteria and concentration in high-volume centers, which may be associated with lower complication rates. Furthermore, evolving surgical techniques, changes in resectoscope design, and increased procedure volumes could have amplified the mechanical and thermal trauma associated with mTURP, contributing to a higher risk of urethral injury. Finally, the broader availability of alternative technologies such as bTURP, HoLEP, and other minimally invasive therapies may have shifted the case mix, with mTURP increasingly reserved for more complex or refractory cases. These factors together may explain the increasingly divergent stricture incidence observed in recent years and highlight the importance of continuous quality monitoring and surgical selection in contemporary practice.

While the study offers valuable insights into the incidence of urethral stricture associated with mTURP and OSP over an extended period, it is important to consider these limitations when interpreting the results. The retrospective design of the study inherently introduces selection bias. An important limitation of our study is the absence of detailed clinical variables, such as preoperative prostate size and postoperative catheterization time. Both factors are known to influence postoperative outcomes and may contribute to the development of urethral strictures. Larger prostates may be associated with longer resection times or more extensive manipulation, potentially increasing the risk of urethral trauma. Similarly, prolonged catheterization has been implicated in the pathogenesis of strictures due to pressure necrosis or infection-related inflammation. Unfortunately, these data were not available in the retrospective database used for this study and therefore could not be incorporated into our multivariable analysis. The omission of these variables may limit the granularity of our findings and should be considered when interpreting the results. Additionally, the absence of preoperative prostate volume estimation poses challenges in assessing how prostate size may impact surgical outcomes or in stratifying patients accordingly. Moreover, the lack of detailed data on comorbidities limits our ability to account for their potential influence on surgical outcomes and mortality rates. Another limitation is the lack of data on the time interval between surgery and the diagnosis of urethral stricture. Consequently, we could not determine the mean time to stricture development in either the mTURP or OSP cohorts. Another limitation of our study is the absence of propensity score analysis, which could have enhanced the adjustment for potential confounding variables between the mTURP and OSP groups. While we employed multivariable Cox proportional hazards models to control for known confounders, such as age, BMI, Charlson comorbidity index, and socioeconomic status, the lack of propensity score methods may limit the robustness of our findings. Future research incorporating propensity score techniques could provide a more comprehensive adjustment for confounding factors and strengthen causal inferences. Furthermore, the unclear documentation regarding indications for surgery hinders our ability to evaluate the appropriateness of treatment choices and outcomes. Significant gaps in our data include the lack of information on prostate-specific antigen (PSA) levels, previous procedures performed, prostate size, median lobe presence, prior history of infections, and pre-existing urethral strictures before surgery. We also do not have details regarding medications used, the precise location, length, manifestation, or timing of urethral strictures, and whether any surgical interventions were required for their management. Additionally, we lack information on preoperative urethral catheterization, catheter indwelling time, and newer treatment modalities such as HoLEP, Rezum, ablation, robotic procedures, and other minimally invasive approaches that have emerged in recent years, including bTURP. These newer procedures could present alternative treatment options with potentially differing efficacy and safety profiles compared to traditional mTURP and OSP.

## 5. Conclusions

Our study highlights a significantly higher incidence of urethral strictures following mTURP compared to OSP. This finding underscores the impact of surgical technique on postoperative stricture formation, perhaps due to direct mechanical injury, trauma, and inflammatory responses associated with mTURP. Moreover, patient factors such as advanced age and higher Charlson comorbidity index scores emerged as independent predictors of stricture development, suggesting that the patient’s overall health and comorbidities play a crucial role in outcomes.

## Figures and Tables

**Table 1:** Demographics and clinical data of patients undergoing mTURP and OSP between 2000 and 2021.

Variable	mTURP (n = 43,525)	OSP (n = 11,347)	*p*-Value
**Age, years (SD)**	73.5 (±9.36)	72.9 (±8.40)	*p* < 0.0001
**BMI, kg/m^2^ (SD)**	27.6 (±4.77)	28.0 (±4.91)	*p* < 0.0001
**Charlson comorbidity index score, n (SD)**	5.51 (±2.81)	4.65 (±2.33)	*p* < 0.0001
**Socioeconomic level, n (%)**			*p* = 0.003
**No data**	1870 (4.2)	449 (3.9)	
**Very low**	1056 (2.4)	263 (2.3)	
**Low**	9856 (22.6)	2765 (24.3)	
**Medium**	15,298 (35.1)	3984 (35.1)	
**High**	11,444 (26.2)	2875 (25.3)	
**Very high**	4001 (9.1)	1011 (8.9)	

mTURP—monopolar TURP; OSP—open simple prostatectomy; BMI—body mass index.

**Table 2:** Multivariable analysis models predicting development of urethral strictures.

Variables	Crude Model			Adjusted Model		
	HR	95%CI	*p*-Value	HR	95%CI	*p*-Value
**Age (years)**	1.032	1.027–1.037	*p* < 0.0001	1.012	1.007–1.018	*p* < 0.0001
**BMI (kg/m^2^)**	0.989	0.981–0.997	*p* = 0.013	0.990	0.982–0.999	*p* = 0.027
**Charlson index score**	1.157	1.141–1.175	*p* < 0.0001	1.128	1.109–1.148	*p* < 0.0001
**Type of surgery (ref. OSP)**	2.467	2.186–2.785	*p* < 0.0001	2.349	2.081–2.653	*p* < 0.0001

HR—hazard ratio; CI—confidence interval; BMI—body mass index; OSP—open simple prostatectomy.

## Data Availability

The data supporting the findings of this study are not publicly available due to privacy and ethical restrictions. The dataset contains sensitive patient information and cannot be shared in accordance with institutional and regulatory guidelines.
